# Expression profile of microRNAs related with viral infectivity, inflammatory response, and immune activation in people living with HIV

**DOI:** 10.3389/fmicb.2023.1136718

**Published:** 2023-03-02

**Authors:** Sara Cuesta-Sancho, Denisse Márquez-Ruiz, Francisco Illanes-Álvarez, Irene Campaña-Gómez, Andrés Martín-Aspas, María Teresa Trujillo-Soto, Alberto Romero, Fátima Galán, Manuel Rodríguez-Iglesias, Mercedes Márquez-Coello, José-Antonio Girón-González

**Affiliations:** ^1^Unidad de Enfermedades Infecciosas, Servicio de Medicina Interna, Facultad de Medicina, Hospital Universitario Puerta del Mar, Instituto de Investigación e Innovación en Ciencias Biomédicas de Cádiz (INiBICA), Universidad de Cádiz, Cádiz, Spain; ^2^Servicio de Microbiología, Facultad de Medicina, Hospital Universitario Puerta del Mar, Instituto de Investigación e Innovación en Ciencias Biomédicas de Cádiz (INiBICA), Universidad de Cádiz, Cádiz, Spain; ^3^Unidad de Enfermedades Infecciosas, Facultad de Medicina, Hospital Universitario Puerto Real, Instituto de Investigación e Innovación en Ciencias Biomédicas de Cádiz (INiBICA), Universidad de Cádiz, Cádiz, Spain

**Keywords:** HIV, people living with HIV, miRNAs, IL-6, sCD163, I-FABP, bacterial translocation

## Abstract

**Objective:**

To evaluate the serum expression of microRNAs (miRNAs) with ability to modulate the human immunodeficiency (HIV) replication or inflammatory status in people living with HIV (PLWH).

**Methods:**

Forty healthy controls and two groups of PLWH were evaluated: (a) Group 1 (*n* = 30), patients with detectable viral load at inclusion, analyzed before receiving antiretroviral therapy (ART) and 12 months after initiating it; (b) Group 2 (*n* = 55), PLWH with prolonged undetectable viral load. Intestinal barrier disruption (I-FABP) and bacterial translocation (16S rDNA) markers, inflammatory markers such as interleukin (IL)-6 and sCD163, immune activation and expression of specific miRNAs were evaluated.

**Results:**

Serum concentrations of I-FABP, 16S rDNA, IL-6, sCD163 and activated T lymphocytes were increased in PLWH. Serum miR-34a was overexpressed at inclusion and remained elevated after ART. The expression of the remaining miRNAs that modulate HIV infectivity (miR-7, mir-29a, miR-150, and miR-223) was similar in PLWH and controls. Related to miRNAs implicated in inflammation (miR-21, miR-155, and miR-210), significant overexpression were observed in miR-21 and miR-210 levels in untreated PLWH, but levels were restored in those patients treated for a long period.

**Conclusion:**

A sustained overexpression of miR-34a was detected even after prolonged HIV controlled replication. miR-21 and miR-210 can be considered new markers of inflammation with high sensitivity to its modifications.

## Introduction

After acute infection by the human immunodeficiency virus (HIV), a depletion of CD4+ T cells in gut-associated lymphoid tissues occurs (Brenchley et al., 2006). The administration of antiretroviral therapy (ART) only partially repairs gut mucosal injury (Jiang et al., 2009). Due to the CD4+ T cell loss and intestinal barrier damage, microbial translocation is detected in HIV-infected patients (Brenchley et al., 2006; Raffatellu et al., 2008; Sandler and Douek, 2012; Hunt et al., 2014). Inflammatory and immune activation are consequences, among others, of the intestinal microbial translocation (Sandler and Douek, 2012; Hunt et al., 2014). Immune activation increases the number of CD4+ T lymphocytes that can be target of HIV (Sandler and Douek, 2012). In addition, persistent inflammation and immune activation have been associated with certain complications in people living with HIV (PLWH), such as metabolic syndrome, type 2 diabetes mellitus or cardiovascular disease (Triant and Grinspoon, 2007).

The inflammatory and immune activation can be modulated, among others, by certain microRNAs (miRNAs) present in PLWH (Sadri Nahand et al., 2020). miRNAs are single-stranded RNAs, of approximately 22 nucleotides, transcribed from DNA genes, but not translated into proteins, whose function is to regulate the expression of other genes. Certain miRNAs can promote (miR-34a) or inhibit (miR-7, miR-29a, miR-150 and miR-223) the replication of HIV in peripheral blood mononuclear cells, promoting the underexpression or overexpression of genes that regulate its cell cycle (Huang et al., 2007; Triboulet et al., 2007; Ahluwalia et al., 2008; Chiang et al., 2012; Fabbri et al., 2012; Duskova et al., 2013; Farberov et al., 2015; Kapoor et al., 2015; Liu et al., 2015; Monteleone et al., 2015; Murray et al., 2015; Hu et al., 2017; Sheng et al., 2017; Bashti et al., 2018). In addition, it has been demonstrated that some miRNAs modulate the increased activation of innate or adaptive immunity (Fabbri et al., 2012; Witwer et al., 2012; Murray et al., 2015; Ruelas et al., 2015; Ballegaard et al., 2017; Jin et al., 2017; Sheng et al., 2017), either by promoting a change in the macrophage phenotype toward an anti-inflammatory state (miR-21; Sheng et al., 2017), decreasing the secretion of proinflammatory cytokines (miR-210; Ballegaard et al., 2017), or by increasing the proportion of regulatory T lymphocytes (miR-155; Witwer et al., 2012). It must be remembered that the immune activation increases the number of CD4+ T lymphocytes that can be targeted by HIV (Sandler and Douek, 2012).

The study of these miRNAs in HIV infection has been carried out mostly in a cross-sectional manner. We hypothesize that changes in the concentration of the miRNAs will correlate with the HIV viral load or with the concentrations of proinflammatory molecules or immune activation before and after ART.

Our objective is to perform a quantitative evaluation of the expression of miRNAs with ability to modulate the HIV replication or inflammatory status in PLWH in different clinical-therapeutic situations: (a) before ART, (b) after 12 months of treatment, when HIV replication is controlled, and (c) in a sample of HIV-infected individuals that had undergone a prolonged time of therapy, to evaluate the long-term consequences of viral suppression on the inflammatory and immune modifications as well as on miRNAs levels. Specifically, the miRNAs studied were those related to infectivity of HIV (miR-34a-activator-, miR-7, miR-29a, miR-150, miR-223-inhibitors; Huang et al., 2007; Ahluwalia et al., 2008; Chiang et al., 2012; Farberov et al., 2015; Kapoor et al., 2015; Monteleone et al., 2015; Hu et al., 2017) or involved in the activation of inflammatory and immune systems (miR-21, miR-155, and miR-210; Fabbri et al., 2012; Witwer et al., 2012; Murray et al., 2015; Ballegaard et al., 2017).

## Patients and methods

### Study design

A prospective, observational study of consecutive cases of HIV infection recruited from a cohort of patients followed up in HIV outpatient clinics at the Puerta del Mar and Puerto Real University Hospitals (Cádiz, Spain) was conducted.

Eighty-five PLWH were divided in two groups: (1) Group 1 included 30 untreated patients with detectable viral load. They were evaluated at the beginning of the study and started ART. They were analyzed just before starting ART and after 12 months with ART. (2) Group 2 included 55 patients with chronic HIV infection and under ART treatment [undetectable HIV loads for a median of 180 months (range, 38–357 months)]. Forty age-and gender-matched healthy controls were recruited.

The exclusion criteria were: (1) acute HIV infection; (2) presence of other opportunistic infections (including viral hepatitis, *Pneumocystis jirovecii*, toxoplasmosis, tuberculosis, cytomegalovirus infections, etc.) or neoplasms. Our screening procedure followed the guidelines established by the Spanish Group for AIDS Study (GeSIDA-Grupo de Estudio de la SEIMC, 2022); (3) active drug use (cocaine, heroin, amphetamines) or significant alcohol ingestion (greater than 50 g/day); (4) treatments that could have modified the determination of inflammation-related molecules or cells (pentoxifylline, anti-inflammatory or immunosuppressive drugs); (5) red blood cell or plasma transfusion in the month before inclusion; (6) non-acceptance of follow-up.

### Definitions

The duration of the HIV infection was established based on the first positive anti-HIV test.

When the HIV load was less than 50 copies/mL (Abbott RealTime HIV-1, Abbott Park, IL, United States), HIV replication was considered to be controlled.

Increased serum concentration of intestinal fatty acid-binding protein (I-FABP) was indicative of gut barrier disruption (Hunt et al., 2014). The bacterial translocation was detected by serum 16S ribosomal DNA (16S rDNA) levels (Abad-Fernández et al., 2013).

Markers of inflammatory activation were serum concentrations of interleukin (IL)-6 and soluble CD163 (sCD163). The activated CD4 and CD8 T lymphocytes were those that simultaneously expressed DR and CD38 on the membrane. Exhausted lymphocytes were those that expressed CD57 and did not express CD28. Finally, those subpopulations that expressed the cell death marker PD1 were analyzed.

### Study schedule

The study protocol included: (1) clinical history, nadir CD4+ T cell count and HIV load, record of previous ART and time with undetectable HIV load in the cases in which it applies; (2) CD4+ T cell count and HIV load at inclusion in the study, (3) peripheral blood sampling at inclusion for analysis of miRNAs, bacterial translocation and inflammatory and immune parameters.

Comparison of results of cell markers and molecules between healthy controls and Group 1 patients (at inclusion) was performed.

In patients with detectable HIV loads at baseline (Group 1), ART was initiated according to the Spanish Group for AIDS Study guidelines (GeSIDA-Grupo de Estudio de la SEIMC, 2022). These patients were followed for 12 months. Bacterial translocation markers, inflammatory and immune parameters and miRNAs were analyzed just before initiation of ART and after 12 months of ART. An index was calculated to quantify the percentage of increase or decrease of molecules after 12 months of ART:

100 × (Molecule concentration after 12 months of ART – Molecule concentration at baseline)/Molecule concentration at baseline.

Current ART was continued in those patients with undetectable HIV loads at inclusion (Group 2). Study parameters were determined only at inclusion.

Comparison of results of cell markers and molecules between Group 1 patients after 12 months of treatment (when undetectable HIV viral load had been obtained) and Group 2 individuals (with a longer period of undetectability) was performed.

### Laboratory methods

Serum was obtained by centrifugation at 1500 g during 15 min at room temperature of blood samples in pyrogen-free heparinized tubes (Biofreeze, Costar, United States), and it was subsequently frozen down at-80°C until its use.

16S rDNA was detected after an initial extraction of DNA (QIAamp DNA Mini Kit; QIAgen, Hilden, Germany) and quantification by spectrophotometry (BioRad, Hercules, CA, United States), as previously described (Márquez-Coello et al., 2021).

Quantikine Human Immunoassays (R&D, Minneapolis, MN, United States) were used to quantify serum concentrations of I-FABP, IL-6 and sCD163, following the indications of the manufacturer.

Fresh blood samples from pyrogen-free heparinized tubes with EDTA (Biofreeze, Costar, United States) were used for flow cytometry. Activated CD4+ and CD8+ T lymphocytes (CD4+ and CD8+ DR+CD38+), senescent status (CD4+ and CD8+ CD57+CD28-) and predisposed to death (CD4+ and CD8+ PD1+) were characterized, as previously described (Márquez-Coello et al., 2021). The stained cells were acquired and analyzed in a BD FACSCanto™ II Cell cytometer, using BD FACSDiva™ Software (BD Biosciences, San Jose, CA, United States). In each case, dead cells were excluded and 300,000 cells were acquired. Blank tubes with no antibodies were used to confirm the specificity of the staining and discriminate the sample from the background.

RNA from serum was isolated using the miRNeasy Serum/Plasma Advanced Kit (QIAGEN, Hilden, Germany), following the instructions of the manufacturer. It was subsequently retrotranscripted with the miRCURY LNA RT Kit (QIAGEN, Hilden, Germany), according to the manufacturer. They were amplified by qPCR, using SYBR® Green master mix and specific LNA primers for miRNAs of interest in the CFX 96 Real-Time System (BioRad, Hercules, CA, United States) thermocycler. Primers for the spike-ins UniSp2 and UniSp5 were used as controls for the RNA extraction. Primers for the spike-in UniSp6 were used as controls for the retrotranscription. Hsa-miR-103a-3p was used as a positive control and data were normalized to this control (Ruiz-de-León et al., 2019). Expression levels of the individual miRNAs were determined by 2^−ΔΔCt^ (Livak and Schmittgen, 2001). All amplifications were done in triplicate. Results were shown as relative units (RU).

### Statistical analysis

Data were expressed as absolute numbers (percentage) or as median values [25–75 interquartile range (IQR)]. Categorical variables were compared using the chi-square test or Fisher’s exact test. The Mann–Whitney U test or ANOVA was used to compare quantitative variables from two independent groups. For comparison of three or more independent groups, Kruskal-Wallis test was used. Friedman’s or Wilcoxon’s rank tests were used to perform paired analysis of variables. The association between quantitative variables was analyzed using the Spearman correlation test. A two-tailed *p* value of <0.05 was considered to be significant. SPSS 22.0 statistical software package (SPSS Inc., Chicago, IL, United States) was used to perform statistical analysis.

### Ethical aspects

This study was performed according to the Helsinki Declaration. The Comité Coordinador de Ética de la Investigación Biomédica de Andalucía (Spain) approved the project (PI-0128-2018-2, Consejería de Salud de la Junta de Andalucía, Spain). Each participant gave written informed consent.

## Results

The demographic, immune and virological characteristics of PLWH (Groups 1 and 2) and controls are shown in Table 1.

**Table 1:** Hematological, immune, and virological characteristics of healthy controls and HIV-infected patients.

	(1)	(2)	(3)	*p* 1 vs. 2	*p* 1 vs. 3	*p* 2 vs. 3
	Healthy controls (*n* = 40)	Chronic HIV-infected patients with detectable HIV load at baseline (Group 1; *n* = 30)	Chronic HIV-infected patients with suppressed HIV load at inclusion (Group 2; *n* = 55)			
Age (years)	43 (30–53)	41 (34–51)	47 (40–56)	0.962	0.180	0.067
Sex male (*n*, %)	34 (85)	27 (90)	46 (84)	0.797	1.000	0.632
Time from HIV diagnosis (months)		2 (1–61)	180 (117–285)			<0.001
CDC stage C (*n*, %)		4 (13)	14 (25)			0.303
**Hematological variables**						
CD4+ T cell/mm3 at diagnosis		456 (210–639)	382 (163–645)			0.307
CD4+ T cells/mm3 at inclusion	886 (580–1,071)	546 (227–659)	771 (529–1,137)	<0.001	0.457	<0.001
CD8+ T cells/mm3 at inclusion	498 (416–662)	1,089 (827–1,379)	917 (658–1,380)	<0.001	<0.001	0.197
**Virological variable**						
HIV load (copies/mL) at inclusion		77,600 (24150–317,000)	<50 (<50–<50)			<0.001

Data are provided as absolute number (percentage) or as median (interquartile range).

### Comparative analysis of untreated people living with HIV and healthy controls

Serum concentrations of I-FABP, 16S rDNA, IL-6, and sCD163 were significantly higher in untreated PLWH with respect to healthy controls. The activation of CD4+ and CD8+ T cells (as measured by membrane expression of CD38 and HLA-DR antigens) and the percentage of those expressing death receptor (PD1) were significantly increased in PLWH with respect to healthy controls (Table 2).

**Table 2:** Gut barrier status, bacterial translocation and immune characteristics of healthy controls and patients with HIV infection, untreated at baseline, analyzed at inclusion and after 12 months of antiretroviral therapy, and of chronically treated HIV patients with undetectable HIV load at inclusion.

	Healthy controls (*n* = 40)	Chronic HIV-infected patients with detectable HIV load at baseline (Group 1; *n* = 30)		Chronic HIV-infected patients with suppressed HIV load at inclusion (Group 2; *n* = 55).	*p* 1 vs. 2	*p* 1 vs. 3	*p* 1 vs. 4	*p* 2 vs. 3	*p* 3 vs. 4
		Prior to antiretroviral therapy	After 12 months of antiretroviral therapy						
	(1)	(2)	(3)	(4)					
**Intestinal barrier and bacterial translocation**									
I-FABP (ng/mL)	782 (590–1,051)	1,111 (793–1,546)	1,058 (674–1709)	1,318 (1034–3,015)	0.019	0.028	<0.001	0.274	0.082
16S rDNA (copies/mL)	2,193 (2014–2,537)	4,212 (3731–4,428)	3,734 (2552–4,920)	2,620 (2186–4,230)	<0.001	<0.001	0.008	0.102	0.236
**Pro-inflammatory molecules**									
Serum IL-6 (pg/mL)	1.0 (0.3–1.5)	4.9 (3.2–8.4)	3.1 (1.1–3.6)	2.7 (1.8–6.8)	<0.001	<0.001	<0.001	0.003	<0.001
Serum soluble CD163 (ng/mL)	476 (352–604)	1,165 (895–1,406)	766 (501–998)	528 (399–734)	<0.001	<0.001	0.150	<0.001	0.006
**Lymphocyte-related parameters**									
CD4+ T cells (percentage of T cells)	46 (39–54)	21 (15–34)	32 (25–39)	29 (22–36)	<0.001	<0.001	<0.001	0.021	0.375
CD4+DR+CD38+ (percentage of CD4+T cells)	1.4 (0.8–1.7)	8.8 (2.5–15.4)	2.5 (1.8–6.6)	2.4 (1.3–4.4)	<0.001	<0.001	<0.001	0.028	0.209
CD4+CD28-CD57+ (percentage of CD4+T cells)	1.2 (0.1–3.3)	8.4 (2.6–14.1)	3.9 (1.8–11.0)	1.8 (0.6–3.8)	<0.001	0.002	0.064	0.507	0.034
CD4+PD1+ (percentage of CD4+T cells)	9.8 (4.2–15.0)	30.9 (14.5–49.7)	22.5 (13.9–26.9)	5.7 (3.0–15.9)	<0.001	<0.001	0.549	0.136	0.001
CD8+ T cells (percentage of T cells)	29 (25–34)	55 (40–66)	42 (37–55)	34 (26–44)	<0.001	<0.001	0.033	0.015	0.521
CD8+DR+CD38+ (percentage of CD4+T cells)	1.9 (1.4–5.1)	22.4 (12.1–29.5)	5.8 (3.4–11.2)	2.5 (1.4–8.1)	<0.001	<0.001	0.485	0.012	0.002
CD8+CD28-CD57+ (percentage of CD4+T cells)	20.1 (11.8–30.3)	16.3 (7.1–33.8)	35.8 (30.4–45.9)	24.1 (17.0–35.5)	0.591	0.001	0.245	0.008	0.005
CD8+PD1+ (percentage of CD4+T cells)	14.3 (8.4–23.6)	29.4 (16.8–49.2)	20.5 (15.8–28.2)	8.6 (2.4–14.5)	<0.001	0.030	0.080	0.023	<0.001

I-FABP, Intestinal fatty acid binding protein; IL-6, interleukin 6. Data are shown as median (interquartile range).

Expression of miR-34a, a miRNA related with facilitation of HIV infection, was significantly higher in untreated PLWH compared with controls. On the contrary, levels of those related with inhibition of HIV replication (miR-7, miR-29a, miR-150, and miR-223) were similar in PLWH and controls (Table 3). There was no significant correlation between the values of these miRNAs and the viral load of the PLWH (data not shown).

**Table 3:** Quantification of the miRNAs involved in the inhibition or facilitation of HIV infection, and of modulation of inflammation and immune activation, in healthy controls and patients with HIV infection, untreated at baseline, analyzed at inclusion and after 12 months of antiretroviral therapy, and of chronically treated HIV patients with undetectable HIV load at inclusion.

	Healthy controls (*n* = 40)	Chronic HIV-infected patients with detectable HIV load at baseline (Group 1; *n* = 30)		Chronic HIV-infected patients with suppressed HIV load at inclusion (Group 2; *n* = 55)	*p* 1 vs. 2	*p* 1 vs. 3	*p* 1 vs. 4	*p* 2 vs. 3	*p* 3 vs. 4
		Prior to antiretroviral therapy	After 12 months of antiretroviral therapy						
	(1)	(2)	(3)	(4)					
**miRNAs involved in the HIV replication**									
miR-34a (RU)	1.0 (1.0–1.0)	1.8 (0.5–3.7)	2.1 (0.5–3.8)	1.6 (1.1–4.8)	0.022	0.030	<0.001	0.557	0.582
miR-7 (RU)	1.0 (1.0–1.0)	1.0 (0.4–2.1)	0.8 (0.4–1.5)	1.2 (0.8–2.3)	0.470	0.397	0.177	0.765	0.058
miR-29a (RU)	1.0 (1.0–1.0)	1.2 (0.6–1.7)	1.2 (0.7–2.0)	1.5 (0.8–2.3)	0.268	0.297	0.091	0.783	0.180
miR-150 (RU)	1.0 (1.0–1.0)	0.8 (0.3–1.5)	1.0 (0.4–2.4)	0.7 (0.4–1.6)	0.171	0.353	0.412	0.112	0.342
miR-223 (RU)	1.0 (1.0–1.0)	1.2 (0.8–2.3)	1.7 (0.5–3.0)	0.9 (0.5–2.4)	0.204	0.425	0.329	0.848	0.562
**miRNAs involved in inflammatory and immune activation**									
miR-21 (RU)	1.0 (1.0–1.0)	2.2 (1.3–3.6)	1.5 (1.0–2.4)	1.1 (0.8–1.6)	<0.001	0.001	0.311	0.010	0.035
miR-155 (RU)	1.0 (1.0–1.0)	1.3 (0.7–2.5)	1.1 (0.7–1.7)	0.9 (0.6–1.9)	0.121	0.787	0.699	0.394	0.842
miR-210 (RU)	1.0 (1.0–1.0)	2.7 (1.2–5.0)	3.4 (0.8–5.1)	0.9 (0.4–1.9)	<0.001	0.011	0.311	0.578	0.002

Data are shown as median (interquartile range) of units relative to healthy control results. RU, Relative units.

**Figure 1:**
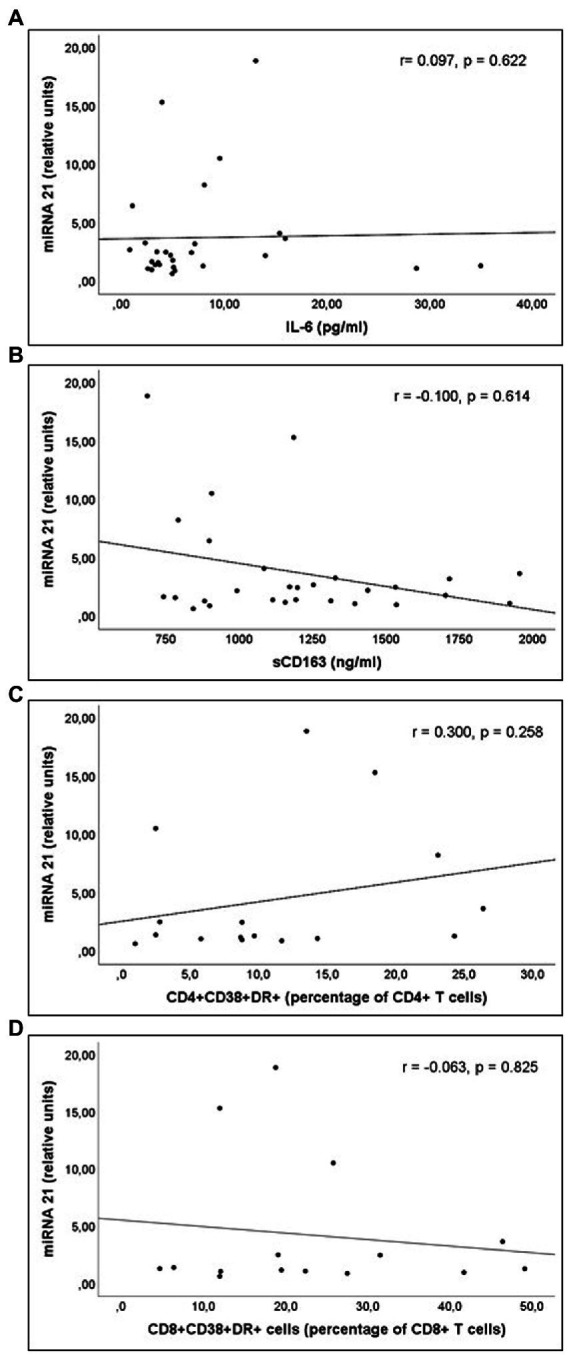
Correlations of miR-21 levels and **(A)** interleukin 6 (IL-6) levels; **(B)** soluble CD163 (sCD163) levels; **(C)** CD4+CD38+DR+ percentage of CD4+ T cell lymphocytes; and **(D)** CD8+CD38+DR+ percentage of CD8+ T cell lymphocytes count, in untreated people living with HIV.

**Figure 2:**
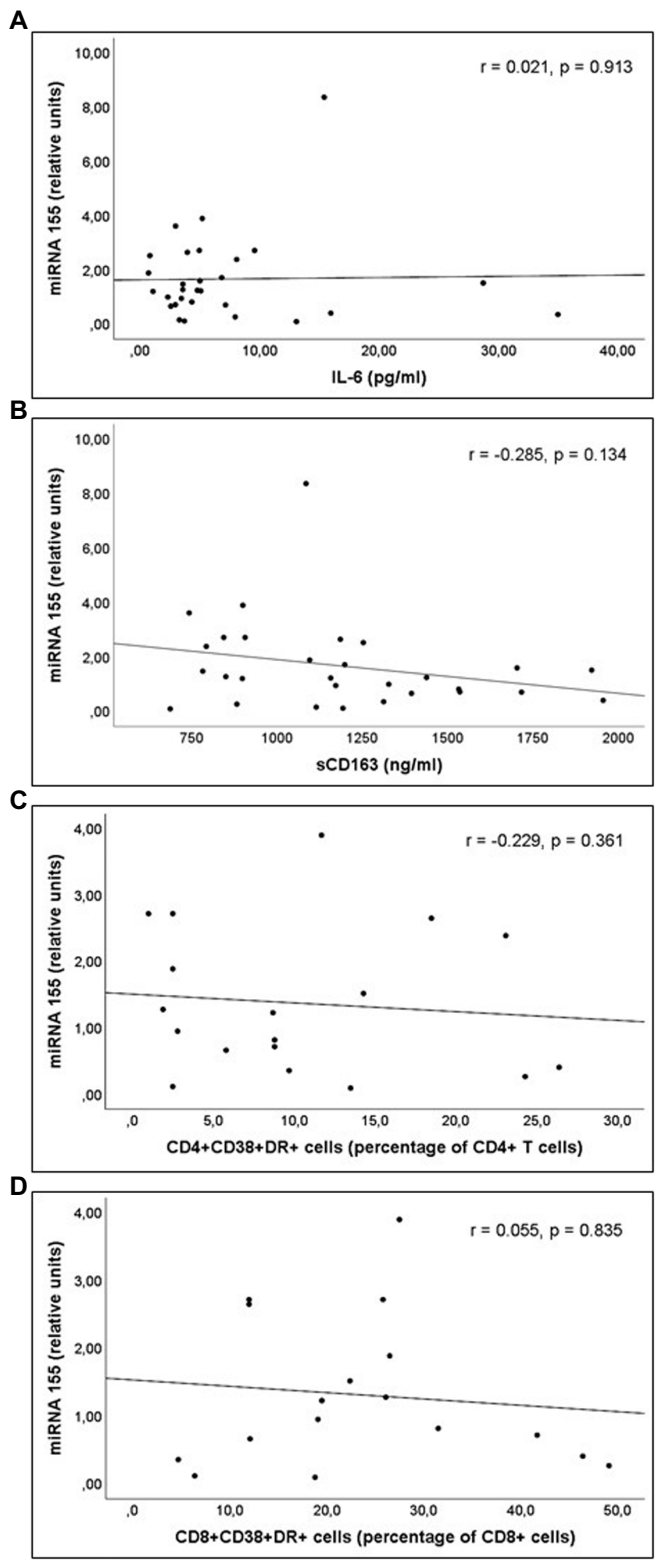
Correlations of miR-155 levels and **(A)** interleukin 6 (IL-6) levels; **(B)** soluble CD163 (sCD163) levels; **(C)** CD4+CD38+DR+ percentage of CD4+ T cell lymphocytes; and **(D)** CD8+CD38+DR+ percentage of CD8+ T cell lymphocytes count, in untreated people living with HIV.

**Figure 3:**
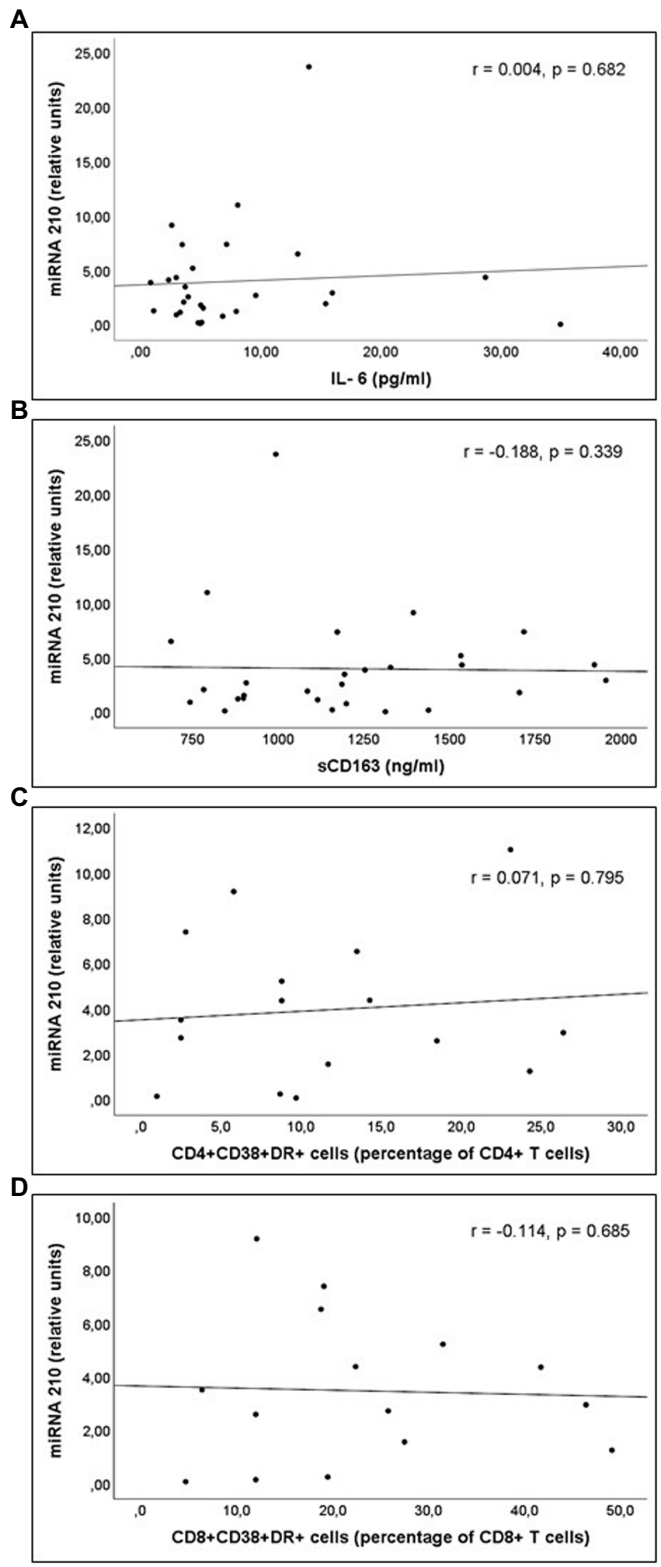
Correlations of miR-210 levels and **(A)** interleukin 6 (IL-6) levels; **(B)** soluble CD163 (sCD163) levels; **(C)** CD4+CD38+DR+ percentage of CD4+ T cell lymphocytes; and **(D)** CD8+CD38+DR+ percentage of CD8+ T cell lymphocytes count, in untreated people living with HIV.

miR-21 and miR-210 levels, implicated in the inflammatory activation, were significantly higher in PLWH than in healthy controls (Table 3). In PLWH, no significant correlation was detected between the values of miRNAs involved in inflammatory or immune regulation and CD4 values (nadir or at the time of inclusion) or viral load. Likewise, there was no significant correlation between the values of these miRNAs and the concentrations of IL-6, sCD163 or the count of activated T lymphocytes (CD4+DR+CD38+ and CD8+DR+CD38+; Figures 1–3).

### Evolution of gut barrier alteration, bacterial translocation, immune markers, and modulating miRNAs in group 1 patients after antiretroviral therapy

Group 1 patients started ART and achieved undetectable HIV loads at 6 months. They were treated either with tenofovir alafenamide and emtricitabine (24 cases) or abacavir and lamivudine (6 cases) associated with integrase inhibitors (24 cases), darunavir/cobicistat (5 cases) or rilpivirine (1 case; Table 2).

During the follow-up, I-FABP and 16S rDNA concentrations remained elevated, without significant differences when compared with baseline values. IL-6 and sCD163 serum concentrations decreased significantly during the follow-up, although they remained elevated relative to healthy controls. CD4+ and CD8+ activated cells (CD38+DR+), as well as CD8+CD28-CD57+ and CD8+PD1+, significantly decreased after 12 months. The proportions of CD4+ expressing PD1 or CD28-CD57+ remained similar during the follow-up (Table 2).

**Figure 4:**
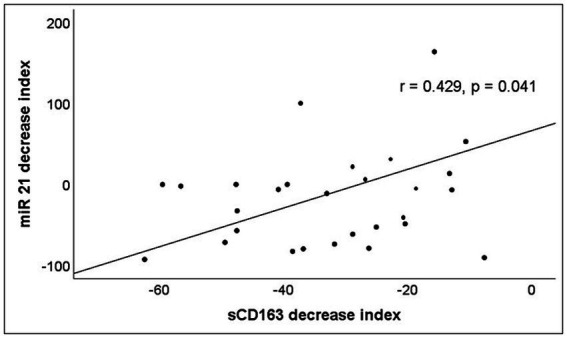
Correlation between the percentage of increase or decrease of serum levels of miR-21 and sCD163 after 12 months of ART. The decrease index was calculated according to the following formula: 100 x (Molecule concentration after 12 months of ART – Molecule concentration at baseline)/Molecule concentration at baseline.

The analysis of miRNAs demonstrated that only miR-21 significantly decreases during the follow-up (Table 3). A significant correlation between the decrease of miR-21 and that of CD163 was observed (Figure 4).

### Comparison of characteristics of HIV-infected patients (group 1) after antiretroviral therapy during 12 months and patients with chronically undetectable HIV load (group 2)

Characteristics observed after 12 months of ART in the 30 HIV-infected individuals that had been untreated at baseline (Group 1) were compared with those of 55 chronically infected patients with undetectable HIV at inclusion as a consequence of previous ART (Group 2) [undetectable HIV load for a median of 180 (range, 38–357) months] (Table 1). Group 2 individuals were being treated either with tenofovir (disoproxil or alafenamide) and emtricitabine (47 patients) or abacavir and lamivudine (8 patients) combined with integrase inhibitors (27 patients), darunavir/cobicistat (15 patients) or rilpivirine (13 patients).

Both I-FABP and 16S rDNA were similar in Group 2 patients when compared to Group 1 individuals treated for only 12 months. A significantly lower serum concentration of IL-6 and sCD163 was observed in Group 2 patients compared to Group 1 subjects treated with ART. Serum I-FABP, 16S rDNA and IL-6 levels in Group 2 patients were significantly higher than those of healthy controls (Table 2).

Group 2 patients showed an increase in the percentage of activated CD4+ T cells with respect to healthy controls. CD4+CD28-CD57+, CD4+PD1+, CD8+CD38+DR+, CD8+CD28-CD57+, and CD8+PD1+ proportions were similar in Group 2 patients and in healthy controls; these values were significantly lower in PLWH with undetectable HIV at inclusion (Group 2) compared with those treated for 12 months (Group 1).

There was no significant difference in the levels of miRNAs implicated in HIV replication between PLWH with undetectable HIV at inclusion (Group 2) compared with those treated for 12 months (Group 1). miR-34a levels were significantly higher in Group 2 patients compared with healthy controls. Analysis of miRNAs implicated in the regulation of inflammatory response demonstrated that significant differences were found in miR-21 and miR-210 expression: their levels were significantly higher in PLWH treated during only 12 months (Group 1) compared with those of Group 2. miR-21 and mir-210 concentrations were similar in healthy controls and Group 2 PLWH.

**Figure 5:**
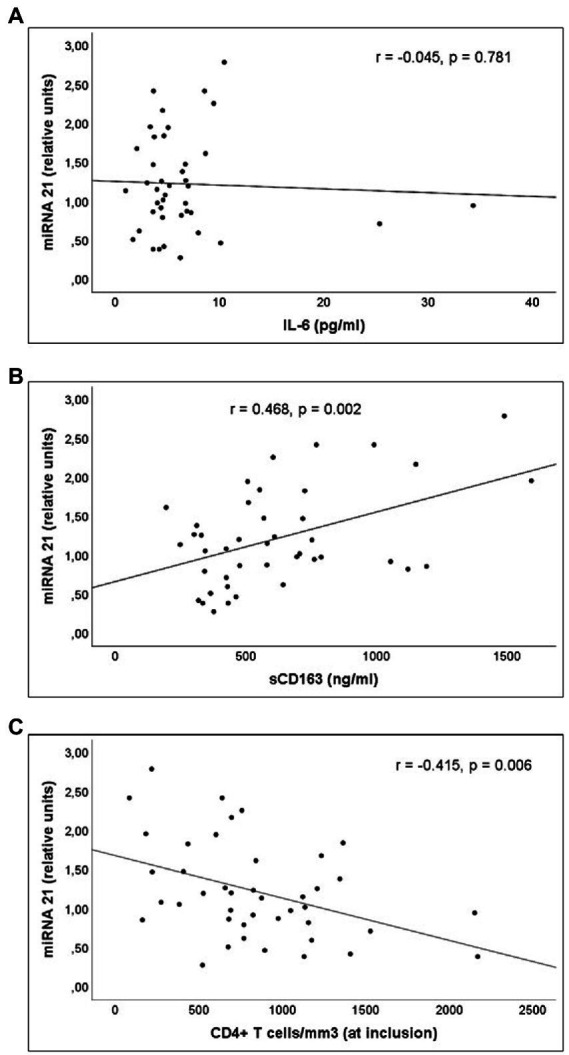
Correlations of miR-21 levels and serum concentration of **(A)** interleukin (IL)-6, **(B)** sCD163 and **(C)** CD4+ T cells/mm3 count at inclusion, in Group 2 patients (PLWH with prolonged undetectable viral load).

**Figure 6:**
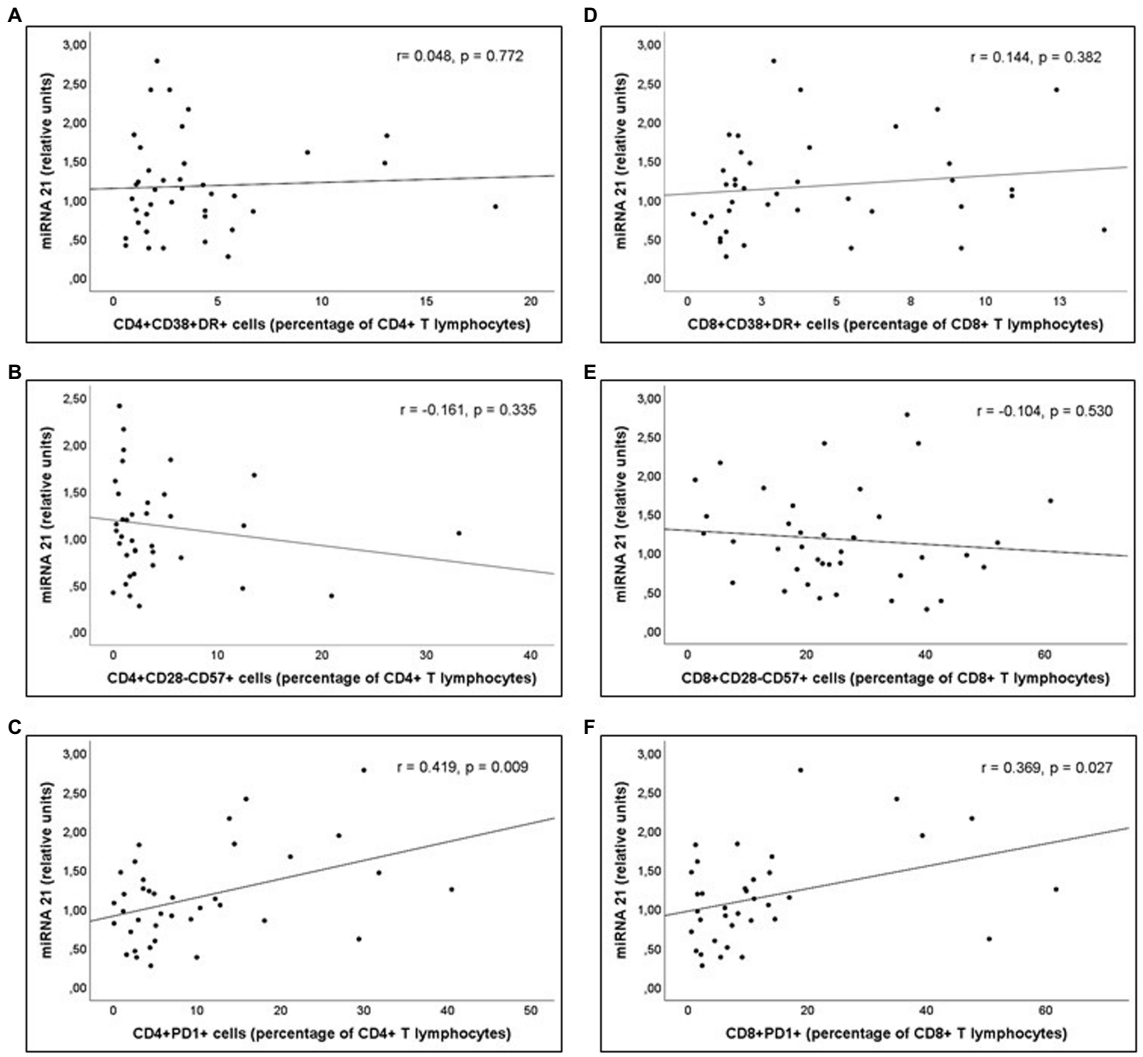
Correlations of miR-21 levels and **(A)** Percentage of CD4+DR+CD38+ (CD4+ T activated lymphocytes), **(B)** Percentage of CD4+CD28-CD57+ (“exhausted” CD4+ T lymphocytes), **(C)** Percentage of CD4+PD1+ (“predisposed to death” CD4+ T lymphocytes), **(D)** Percentage of CD8+DR+CD38+ (CD8+ T activated lymphocytes), **(E)** Percentage of CD8+CD28-CD57+ (“exhausted” CD8+ T lymphocytes), and **(F)** Percentage of CD8+PD1+ (“predisposed to death” CD8+ T lymphocytes), in Group 2 patients (PLWH with prolonged undetectable viral load).

Analysis of the correlations between the parameters indicative of inflammatory or immune activation and the miRNAs involved in these responses was performed in PLWH with prolonged undetectable viral load (Group 2). A statistically significant correlation was observed between serum miR-21 expression and concentrations of sCD163, as well as between miR-21 expression and CD4+ T lymphocytes/mm^3^ values at the time of inclusion (Figure 5). Likewise, a significant correlation was observed between serum miR-21 levels and percentages of CD4+PD1+ and CD8+PD1+ cells (Figure 6).

In PLWH with prolonged undetectable viral load (Group 2), the influence of ART on inflammatory or immune activation parameters together with the miRNAs expression was performed. No significant differences were observed in these parameters depending on the different ART treatments [tenofovir plus emtricitabine or abacavir plus lamivudine (Supplementary Table 1)]. Similarly, no statistically significant differences were found in these values depending on whether the third drug was a non-nucleoside reverse transcriptase inhibitor, an integrase inhibitor, or a viral protease inhibitor (Supplementary Table 2).

## Discussion

The present study has shown the existence of a permanent alteration of intestinal permeability, bacterial translocation and inflammatory and immune activation in PLWH. The analysis of the miRNAs reveals the existence of high values of some of those involved in facilitating HIV infection and in modulating inflammatory activation.

As it has previously been communicated (Brenchley et al., 2006; Jiang et al., 2009), intestinal barrier alteration (assessed by increased concentration of I-FABP) and microbial translocation (assessed by the elevated levels of 16S rDNA in serum), as well as inflammatory (measured by IL-6 or sCD163 levels) and T lymphocytes activation (CD4+DR+CD38+ and CD8+DR+CD38+ percentages) were detected in untreated PLWH. The proportion of T lymphocytes exhausted or expressing death receptors were also significantly elevated in these individuals. After controlling viral replication by ART, and even when bacterial translocation is not significantly modified, there was a decrease in inflammatory and immune activation, although it persisted at values higher than those detected in healthy controls.

This study has analyzed the role of miRNAs that could modify the infective capacity of HIV or modulate the inflammatory and immune activation of these individuals. The selected miRNAs have been those that have shown the ability to influence any of these phases, according to existent literature (Huang et al., 2007; Ahluwalia et al., 2008; Chiang et al., 2012; Fabbri et al., 2012; Witwer et al., 2012; Kapoor et al., 2015; Monteleone et al., 2015; Murray et al., 2015; Ballegaard et al., 2017).

miR-34a expression, but not that of miR-7, miR-29a, miR-150 or miR-223, was increased in untreated PLWH compared with controls. A positive feedback between miR-34a and HIV has been demonstrated: HIV induces the expression of miR-34a in T lymphocytes and miR-34a favors the replication of HIV in these cells, inhibiting either the phosphatase 1 nuclear-targeting subunit (Kapoor et al., 2015), the histone deacetylase sirtuin 1 (Hu et al., 2017) or the human potassium channel protein TASK-1 (Farberov et al., 2015), that regulate negatively HIV transcription. The miR-34a is significantly upregulated in mononuclear cells of PLWH with high viral load (Duskova et al., 2013). Our data support such a state of upregulation by providing evidence of increased peripheral blood miR-34a levels in PLWH. Additionally, we have been able to verify that the overexpression of this miRNA persists even after controlling viral replication by ART for very long periods. It is possible to speculate that, even when these patients had an undetectable HIV viral load in peripheral blood, the virus could be active in some other location (mainly lymph nodes or gut-associated lymphoid tissue) and the stimulus for the synthesis of miR-34a persisted. Furthermore, this suggests the existence of a favorable state for virus replication in case the ART blockade disappears. Our data open a new line toward the possibility of drug treatments directed against miR-34a in conjunction with ARTs. On the other hand, miR-34a has been implicated in endothelial cell senescence and vascular aging in PLWH (Zhan et al., 2016). The persistence of an overexpression of miR-34a, even after long periods of viral undetectability, could justify the demonstrated higher prevalence of cardiovascular disease in PLWH (Gibellini et al., 2013).

Several miRNAs (miR-7, miR-29a, miR-150, and miR-223) have enriched expression in resting primary CD4+T lymphocytes and have direct target sites on HIV-1 mRNA, suggesting that they could influence resistance against HIV (Huang et al., 2007). miR-7 may be induced in response to infections of several mammalian viruses, although the mechanisms through which it exerts its effect are not clarified (Zhang et al., 2015). miR-29 downregulates HIV-1 Nef transcripts and Nef protein expression, as well as Tat-dependent transactivation of viral gene expression (Chiang et al., 2012). miR-150 could influence HIV replication by targeting a cellular cofactor (CCNT1) *via* c-Myb (Huang et al., 2007). miR-223 attenuates viral gene expression by directly targeting HIV-1 mRNAs in dormant CD4+ T cells where HIV replication is silenced (Yuan et al., 2021). Upregulation of these miRNAs leads to an inhibition of HIV replication (Leo et al., 2021).

The overexpression of these miRNAs has been demonstrated in different cell types (Wang et al., 2009; Munshi et al., 2014; Narla et al., 2018; Biswas et al., 2019), although the results in this regard are controversial (Wang et al., 2009; Munshi et al., 2014; Liu et al., 2015; Bazié et al., 2022). An overexpression of miR-7 (Biswas et al., 2019), miR-29 (Narla et al., 2018), miR-150 (Munshi et al., 2014) and miR-223 (Munshi et al., 2014; Narla et al., 2018) has been observed in the PLWH plasma by some authors, while others have detected values similar to those of healthy controls (Bazié et al., 2022) or, when analyzed in cell populations, decreased (Wang et al., 2009; Munshi et al., 2014; Liu et al., 2015). It is possible that these discrepancies, as well as the differences of some of them with our results, are due to the characteristics of the patients included, specifically derived from their viral load or CD4+ T lymphocyte counts, or to the inclusion of treated and untreated patients, without differentiation between them. Our data show that expression of miR-7, −29a, −150, and −223 are similar in viremic patients and healthy controls, and that these serum levels do not change after a short or long period of therapy. These data argue against an important modulation of the viral infectivity by these miRNAs in patients with HIV infection.

miRNAs inhibit HIV-1 expression by either directly interfering with viral mRNAs or by modulating host innate immunity. IL-6 stimulates the expression of CD163 molecules (Fabriek et al., 2005) and thus sCD163 concentrations can be considered, at least in part, a consequence of IL-6-promoted proinflammatory activation. In our study, IL-6, sCD163 and activated T lymphocytes were selected as markers of inflammatory or immune activation.

miR-155 controls differentiation of CD4^+^ T cells into the T helper type 1 (Th1), Th2, and Th17 subsets of T helper cells and it affects the development of T regulatory (Treg) cells (Seddiki et al., 2014). miR-155 levels are increased after *in vitro* macrophage stimulation of Toll-like receptors 3 and 4 (Witwer et al., 2012). High levels of miR-155 have been found previously in total PBMCs, CD4 T cells and CD8 T cells in PLWH (Jin et al., 2017; Zhang et al., 2021). In our series, miR-155 level was similar in controls and in PLWH, treated or untreated. It is possible that the difference with other authors is due to the fact that in our study the levels observed were those of serum and not those of PBMCs. In any case, the absence of correlation with activation parameters and the absence of significant change in serum levels after ART is significant.

The expression of miR-210 is induced by LPS in macrophages. This miRNA works as a negative regulator for LPS-induced expression of proinflammatory cytokines – it targets NF-κB1 to decrease NF-κB activation (Qi et al., 2012). It has been demonstrated that miR-210 was upregulated in PBMCs from treated HIV-infected individuals (Ballegaard et al., 2017). In our case, miR-210 was increased in untreated or treated PLWH for 12 months compared with healthy controls. However, and in the same way as other inflammatory parameters, its concentration was similar to healthy controls in those treated for very long periods.

miR-21 is increased in response to oxidative stress or LPS in macrophages and regulates macrophage functions (Krichevsky and Gabriely, 2009; Thulasingam et al., 2011). miR-21 inhibits inflammation by targeting JAK2/STAT3 pathway (Sheng et al., 2017). Interestingly, it modulates the cytokine output of macrophages, from a proinflammatory to anti-inflammatory profile, suggesting that miR-21 is involved in macrophage polarization (Barnett et al., 2016). Besides its anti-inflammatory role, it has also been hypothesized to be involved in the permeability of the intestinal barrier, since it regulates the intestinal epithelial tight junction permeability through PTEN/PI3K/Akt signaling pathway (Zhang et al., 2015). Increased levels of miR-21 have been observed in treated HIV-infected patients (Chettimada et al., 2020). Our work has shown that miR-21 expression levels are increased in untreated subjects, as do the serum concentrations of IL-6 and sCD163. A short period of treatment (12 months) of previously untreated patients (Group 1) induces a decrease in both IL-6 and sCD163 as well as miR-21, although they remain elevated compared to healthy controls. The decreases (measured as a percentage from baseline values) of sCD163 and miR-21 were positively and significantly correlated. Patients with long-term treatment (Group 2) present values of both sCD163 and miR-21 similar to those of healthy individuals. Consequently, we can affirm that miR-21 is a new marker of inflammatory activation, with high sensitivity to the changes experienced in it.

In the same way as sCD163 and miR-21, there was a significant decrease in the percentages of CD4+PD1+ and CD8+PD1+ (T lymphocytes predisposed to death) in PLWH with a long treatment time (Group 2) compared to those treated with ART for only 12 months (Group 1). In fact, patients of Group 2 show values similar to those of the healthy controls. The relationship of miR-21 with the expression of PD1 receptors in T lymphocytes has not been previously described.

### Limitations

Although each reported anti-HIV miRNA has been validated by one or more research groups, there are no miRNAs universally identified as anti-HIV miRNAs. These studies cannot be compared to each other easily because of the differences in patients’ detection methods and candidate miRNAs studied, as well as in the types of samples.

## Conclusion

In conclusion, the present study has analyzed PLWH in different clinical situations (untreated, after a short period of treatment-12 months-or after a prolonged period of treatment-180 months) and has demonstrated the usefulness of three miRNAs in the follow-up of these individuals: (a) miR-34a, as a marker that possibly implies the ability to reactivate HIV after treatment withdrawal, and (b) miR-21 and miR-210, that would probably act as regulatory factors of inflammatory activation.

## Data availability statement

The original contributions presented in the study are included in the article/Supplementary material, further inquiries can be directed to the corresponding author.

## Ethics statement

The studies involving human participants were reviewed and approved by Comité Coordinador de Ética de la Investigación Biomédica de Andalucía (Spain). The patients/participants provided their written informed consent to participate in this study.

## Author contributions

SC-S, DM-R, MM-C, and J-AG-G conceived and designed the experiments. SC-S, DM-R, FI-Á, IC-G, AM-A, MT-S, AR, FG, and MR-I performed the experiments. SC-S, DM-R, FI-Á, IC-G, MM-C, and J-AG-G analyzed the data. SC-S, DM-R, FI-Á, IC-G, MT-S, FG, and MR-I contributed reagents, materials, and analysis tools. SC-S, DM-R, FI-Á, IC-G, MM-C, and J-AG-G wrote the draft. All authors contributed to conception of the study, and critical revision of the manuscript, and saw and approved the final version.

## Funding

This work was supported by the Consejería de Salud de la Junta de Andalucía, Spain, Iniciativa territorial integrada 2014-2020 para la provincia de Cádiz [No PI-0076-2017]; by the Consejería de Salud de la Junta de Andalucía, Spain, Proyectos de investigación en Salud [No PI-0128-2018]; and by Instituto de Salud Carlos III, Acción Estratégica en Salud 2019 [No PI19/01361], Spain. Co-financed by European Regional Development Fund (FEDER).

## Conflict of interest

The authors declare that the research was conducted in the absence of any commercial or financial relationships that could be construed as a potential conflict of interest.

## Publisher’s note

All claims expressed in this article are solely those of the authors and do not necessarily represent those of their affiliated organizations, or those of the publisher, the editors and the reviewers. Any product that may be evaluated in this article, or claim that may be made by its manufacturer, is not guaranteed or endorsed by the publisher.
