# Supplementary material for: Expression profile of microRNAs related with viral infectivity, inflammatory response, and immune activation in people living with HIV
Source: Front Microbiol. 2023 Mar 2;14:1136718. doi: 10.3389/fmicb.2023.1136718 (PMC10017538; doi:10.3389/fmicb.2023.1136718)
Supplement: Supplementary file 1 [file Table_1.pdf]

SUPPLEMENTARY MATERIAL (Table 1)

TITLE: **Expression profile of microRNAs related with viral infectivity, inflammatory response and immune activation in people living with HIV.**

Short title: **microRNAs in HIV-infected patients.**

AUTHORS: **Sara Cuesta-Sancho [1 †], Denisse Márquez-Ruiz [1 †], Francisco Illanes-Álvarez [1], Irene Campaña-Gómez [1], Andrés Martín-Aspas [1], María Teresa Trujillo-Soto [2], Alberto Romero [3], Fátima Galán [2], Manuel Rodríguez-Iglesias [2], Mercedes Márquez-Coello [1 *], José-Antonio Girón-González [1 *].**

[1]Unidad de Enfermedades Infecciosas, Servicio de Medicina Interna, Hospital Universitario Puerta del Mar, Facultad de Medicina, Universidad de Cádiz, Instituto de Investigación e Innovación en Ciencias Biomédicas de Cádiz (INiBICA), 11009, Cádiz, Spain.

[2]Servicio de Microbiología, Hospital Universitario Puerta del Mar, Facultad de Medicina, Universidad de Cádiz, Instituto de Investigación e Innovación en Ciencias Biomédicas de Cádiz (INiBICA),11009, Cádiz, Spain.

[3]Unidad de Enfermedades Infecciosas, Hospital Universitario Puerto Real, Facultad de Medicina, Universidad de Cádiz, Instituto de Investigación e Innovación en Ciencias Biomédicas de Cádiz (INiBICA),11009, Cádiz, Spain.

[†] These authors contributed equally to this work and share first authorship.

***** These authors contributed equally to this work and share last authorship.

Supplementary Table 1. Intestinal barrier and bacterial translocation, proinflammatory molecules, immune activation and miRNAs in patients living with HIV with undetectable viral load at inclusión (Group 2) in function of the antiretroviral therapy with tenofovir plus emtricitabina vs abacavir plus lamivudine.

Parameter	Patients on treatment with tenofovir plus emtricitabina (n=47)	Patients on treatment with abacavir plus lamivudina (n=8)	p
**Intestinal barrier and bacterial translocation**			
I-FABP (ng/ml)	1492 (908-2666)	1344 (1026-3013)	0.734
16S rDNA (copies/ml)	2006 (1702-4363)	2537 (1811-3944)	0.292
**Pro-inflammatory molecules**			
Serum IL-6 (pg/ml)	2.7 (1.7-6.8)	2.7 (2.1-6.8)	0.903
Serum soluble CD163 (ng/ml)	508 (390-683)	545 (366-771)	0.543
**Lymphocyte-related parameters**			
CD4+ T cells (percentage of T cells)	30 (20-36)	29 (24-36)	0.903
CD4+DR+CD38+ (percentage of CD4+T cells)	3.0 (1.5-4.5)	1.8 (1.1-4.2)	0.354
CD4+CD28-CD57+ (percentage of CD4+T cells)	1.7 (1.4-10.5)	1.2 (0.4-3.2)	0.082
CD4+PD1+ (percentage of CD4+T cells)	4.8 (3.1-15.7)	7.0 (3.0-16.3)	0.138
CD8+ T cells (percentage of T cells)	33 (26-41)	34 (28-46)	0.780
CD8+DR+CD38+ (percentage of CD4+T cells)	2.7 (1.3-4.5)	2.1 (1.4-8.5)	0.557
CD8+CD28-CD57+ (percentage of CD4+T cells)	27.5 (21.1-41.4)	21.1 (11.5-29.7)	0.090
CD8+PD1+ (percentage of CD4+T cells)	7.9 (2.4-9.8)	9.8 (4.2-16.6)	0.294
**miRNAs involved in the HIV replication**			
miR-34a (RU)	1.3 (0.7-4.8)	1.8 (1.2-5.1)	0.659
miR-7 (RU)	1.3 (0.5-2.1)	1.2 (0.8-2.9)	0.529
miR-29a (RU)	1.4 (0.7-2.3)	1.6 (1.0-2.4)	0.488
miR-150 (RU)	0.6 (0.4-1.8)	1.1 (0.4-1.6)	0.683
miR-223 (RU)	1.0 (0.5-3.7)	0.8 (0.4-1.9)	0.699
**miRNAs involved in inflammatory and immune activation**			

miR-21 (RU)	1.0 (0.6-1.3)	1.2 (0.9-1.8)	0.121
miR-155 (RU)	0.9 (0.5-1.6)	0.9 (0.5-2.4)	0.683
miR-210 (RU)	0.7 (0.4-1.1)	1.0 (0.4-2.0)	0.131

Data are shown as median (interquartile range) of units relative to healthy control results.

Abbreviations: RU: Relative units
