# Supplementary material for: Expression profile of microRNAs related with viral infectivity, inflammatory response, and immune activation in people living with HIV
Source: Front Microbiol. 2023 Mar 2;14:1136718. doi: 10.3389/fmicb.2023.1136718 (PMC10017538; doi:10.3389/fmicb.2023.1136718)
Supplement: Supplementary file 2 [file Table_2.pdf]

SUPPLEMENTARY MATERIAL (Table 2)

TITLE: **Expression profile of microRNAs related with viral infectivity, inflammatory response and immune activation in people living with HIV.**

Short title: **microRNAs in HIV-infected patients.**

AUTHORS: **Sara Cuesta-Sancho [1 †], Denisse Márquez-Ruiz [1 †], Francisco Illanes-Álvarez [1], Irene Campaña-Gómez [1], Andrés Martín-Aspas [1], María Teresa Trujillo-Soto [2], Alberto Romero [3], Fátima Galán [2], Manuel Rodríguez-Iglesias [2], Mercedes Márquez-Coello [1 *], José-Antonio Girón-González [1 *].**

[1]Unidad de Enfermedades Infecciosas, Servicio de Medicina Interna, Hospital Universitario Puerta del Mar, Facultad de Medicina, Universidad de Cádiz, Instituto de Investigación e Innovación en Ciencias Biomédicas de Cádiz (INiBICA), 11009, Cádiz, Spain.

[2]Servicio de Microbiología, Hospital Universitario Puerta del Mar, Facultad de Medicina, Universidad de Cádiz, Instituto de Investigación e Innovación en Ciencias Biomédicas de Cádiz (INiBICA),11009, Cádiz, Spain.

[3]Unidad de Enfermedades Infecciosas, Hospital Universitario Puerto Real, Facultad de Medicina, Universidad de Cádiz, Instituto de Investigación e Innovación en Ciencias Biomédicas de Cádiz (INiBICA),11009, Cádiz, Spain.

[†] These authors contributed equally to this work and share first authorship.

***** These authors contributed equally to this work and share last authorship.

Supplementary Table 2. Intestinal barrier and bacterial translocation, proinflammatory molecules, immune activation and miRNAs in patients living with HIV with undetectable viral load at inclusión (Group 2) in function of the antiretroviral therapy with non-nucleoside transcriptase inhibitor, integrase inhibitors or protease inhibitors.

Parameter	(1) Patients on treatment with non-nucleoside transcriptase inhibitor (rilpivirine) (n=13)	(2) Patients on treatment with integrase inhibitors (n=27)	(3) Patients on treatment with protease inhibitors (n=15)	p 1 vs 2	p 1 vs 3	p 2 vs 3
**Intestinal barrier and bacterial translocation**						
I-FABP (ng/ml)	1539 (919-3282)	1686 (1204-3514)	1393 (1132-3017)	0.303	0.614	0.887
16S rDNA (copies/ml)	2636 (2063-4176)	2502 (2186-4260)	2643 (2257-4263)	0.900	0.758	0.701
**Pro-inflammatory molecules**						
Serum IL-6 (pg/ml)	3.5 (3.1-7.7)	2.6 (2.0-6.8)	3.4 (2.4-6.3)	0.745	0.816	0.281
Serum soluble CD163 (ng/ml)	531 (312-652)	570 (426-727)	583 (409-963)	0.127	0.180	0.842
**Lymphocyte-related parameters**						
CD4+ T cells (percentage of T cells)	28 (25-36)	29 (21-36)	32 (19-38)	0.512	0.880	0.754
CD4+DR+CD38+ (percentage of CD4+T cells)	1.6 (0.9-4.0)	2.4 (1.3-4.4)	3.6 (1.8-5.2)	0.328	0.113	0.328
CD4+CD28-CD57+ (percentage of CD4+T cells)	1.2 (0.3-4.4)	2.1 (1.0-4.9)	2.1 (0.6-8.2)	0.214	0.418	0.721
CD4+PD1+ (percentage of CD4+T cells)	4.4 (2.2-10.9)	5.4 (2.9-22.5)	7.1 (3.8-16.0)	0.456	0.270	0.814
CD8+ T cells (percentage of T cells)	31 (26-39)	38 (28-46)	30 (25-44)	0.376	1.000	0.530
CD8+DR+CD38+ (percentage of CD4+T cells)	1.9 (1.5-3.5)	2.5 (1.2-8.7)	2.7 (1.5-7.7)	0.766	0.418	0.766

CD8+CD28-CD57+ (percentage of CD4+T cells)	21.2 (19.1-32.4)	25.4 (8.9-35.4)	25.8 (17.9-45.0)	0.394	0.347	0.422
CD8+PD1+ (percentage of CD4+T cells)	8.8 (3.9-10.4)	8.4 (1.8-17.8)	6.7 (2.8-13.4)	0.926	0.794	0.798
**miRNAs involved in the HIV replication**						
miR-34a (RU)	1.7 (1.3-6.6)	1.2 (0.8-3.5)	2.0 (1.2-3.6)	0.081	0.373	0.311
miR-7 (RU)	1.0 (0.8-1.1)	1.8 (0.8-2.3)	1.2 (0.5-3.0)	0.187	0.829	0.208
miR-29a (RU)	1.4 (1.1-2.4)	1.4 (0.6-2.3)	1.7 (1.1-3.2)	0.658	0.635	0.443
miR-150 (RU)	1.4 (0.2-5.1)	1.1 (0.4-1.6)	0.6 (0.4-1.1)	0.708	0.587	0.749
miR-223 (RU)	1.3 (0.3-4.2)	0.9 (0.5-3.6)	0.8 (0.6-2.4)	0.791	0.875	0.897
**miRNAs involved in inflammatory and immune activation**						
miR-21 (RU)	1.2 (0.5-1.5)	1.2 (0.8-1.8)	1.0 (0.9-1.2)	0.674	0.633	0.237
miR-155 (RU)	1.3 (0.4-2.7)	0.9 (0.5-1.9)	0.9 (0.7-1.1)	0.812	0.562	0.941
miR-210 (RU)	0.9 (0.4-1.9)	0.5 (0.4-1.6)	1.1 (0.6-2.5)	0.947	0.692	0.428

Data are shown as median (interquartile range) of units relative to healthy control results.

Abbreviations: RU: Relative units
